# An investigation of race bias in deep learning-based segmentation of prostate MRI images

**DOI:** 10.1038/s41598-025-26189-5

**Published:** 2025-12-06

**Authors:** Maram Alqarni, Emma-Louise Jones, Luis Ribeiro, Hema Verma, Sian Cooper, Vinod Mullassery, Stephen Morris, Teresa Guerrero Urbano, Andrew P. King

**Affiliations:** 1 School of Biomedical Engineering and Imaging Sciences, Faculty of Life Sciences and Medicine, King’s College London, London, SE1 7EU United Kingdom; 2 Biomedical Engineering Department, Imam Abdurahman bin Faisal University, Dammam, Saudi Arabia; 3 Department of Clinical Haematology and Oncology, Guy’s and St Thomas’ NHS Foundation Trust, Guy’s Cancer Centre, Guy’s Hospital, Great Maze Pond, London, SE1 9RT United Kingdom; 4 Department of Medical Physics and Clinical Engineering, Guy’s Hospital, Great Maze Pond, London, SE1 9RT United Kingdom

**Keywords:** Deep learning, Race, Bias, Prostate, MRI, Radiotherapy, Segmentation, Biomedical engineering, Machine learning, Prostate

## Abstract

Deep learning (DL) has been proposed for magnetic resonance imaging (MRI) prostate segmentation for various clinical tasks, including radiotherapy treatment planning. In other applications, DL models have exhibited performance bias by protected attributes such as race. To investigate possible race bias in prostate MRI segmentation, DL models were trained on five clinical T2-weighted MRI datasets with varying White/Black race imbalance, plus one public dataset with unknown races, and evaluated on 32 White/Black matched clinical subjects. For the models trained with differing levels of race imbalance, the best performance for both races was when the training set was race-balanced. A linear mixed-effects model analysis showed that Dice Similarity Coefficient (DSC) differences between Black and White subjects depended on race representation in the training data, with a slight reduction in White-Black performance gap as Black representation increased (p < 0.05). The model trained on public data showed no difference in performance between races for DSC. The findings reveal the potential for race bias in DL prostate MRI segmentation performance when training sets are highly imbalanced. We argue for transparency in race reporting in DL prostate segmentation training data and reporting of test performance across demographic groups, with appropriate ethical/legal safeguards.

## Introduction

Accurate delineation of the prostate from magnetic resonance imaging (MRI) is important for various clinical applications, including cancer detection/diagnosis and measuring prostate specific antigen density. In prostate radiation oncology, segmentation is particularly crucial for defining the clinical target volume (CTV) and organs at risk (OARs), to ensure the delivery of a precise dose to the CTV while sparing healthy tissue. The transition towards using MRI for radiation treatment planning for prostate cancer can allow clinicians to more accurately segment the CTV and OARs due to its superior soft tissue contrast compared to Computed Tomography (CT)^1,2^. Despite the superior image quality of MRI, manual segmentation of the CTV and OARs suffers from inter and intra-observer variability and is time-consuming, which slows down the treatment pathway and in adaptive radiotherapy workflows, also delivery.

Recently, deep learning (DL) has been proposed to automate prostate MRI segmentation^2–12^. For example, a recent review by Mohammad et al. highlighted the growing use of DL for MRI prostate segmentation, with a notable peak in publications between 2019 and 2021^13^. Specifically, convolutional neural networks (CNNs) have been the most common type of DL for image-based segmentation. For example, the nnU-Net^14^ framework is an automated pipeline that adjusts preprocessing, architecture tuning, training, and postprocessing for a standard U-Net CNN^15^. Due to its consistently competitive performance across a range of applications, including prostate MRI, this architecture was chosen as the main pipeline used in this work.

However, DL models can be sensitive to differences in the image characteristics between the data used for model training and those used during model deployment. These differences (or *distributional shifts*) can arise due to various reasons such as MRI scanner vendor/field strength, pathology and patient demographics, and can lead to poorer performance on certain groups of data when the model is deployed, a phenomenon known as model bias^16^. Several studies have shown that imbalances in patient demographics, e.g. age, gender, ethnicity/race or socioeconomic status can propagate bias into DL based classification models, which can in turn impact health outcomes for under-represented groups^17–20^. In DL-based medical image segmentation, biases have been found due to race/sex in cardiac MRI segmentation^21–25^, race/sex in brain MRI segmentation^26^, skin tone in skin lesion segmentation^27^ and race/sex/age in orthopedic X-ray segmentation^28^. However, no work has yet investigated the possibility of race bias in DL-based prostate MRI segmentation.

In this paper, we perform such a study. First, we investigate the potential for race bias between Black and White subjects in a controlled experimental setting using MRI data acquired for clinical purposes, by systematically varying levels of race imbalance in training datasets and evaluating the trained models on matched pairs of Black and White subjects. Second, we investigate whether race bias can occur when training on a large amount of publicly available MRI data from subjects with unknown race, again evaluating the model on clinical MRIs containing paired Black and White race subjects.

## Methods

### Datasets

For the first experiment (investigating the impact of training set race imbalance), we employed an institutional clinical dataset retrieved from the Picture Archiving and Communication System (Sectra PACS) of Guy’s and St. Thomas’ NHS Foundation Trust (GSTFT). This project was carried out under the Guy’s cancer cohort with an ethical approval obtained from Health Research Authority North West—Haydock Research Ethics (REC Reference:18/NW/0297, IRAS project ID 231443.). The need for informed consent was waived off by the committee because this is a research database held by GSTFT that operates an “opt out” consent process. Access to the data is via an institutional data access committee that approves all research projects internally. No patients included in this study opted out of the Guy’s Cancer cohort. The research was carried out in accordance with the Declaration of Helsinki, the Early Value Assessment guidance of the UK National Institute for Health and Care Excellence^29^ and the Tripod + AI Guidelines^30^.

**Fig. 1:**
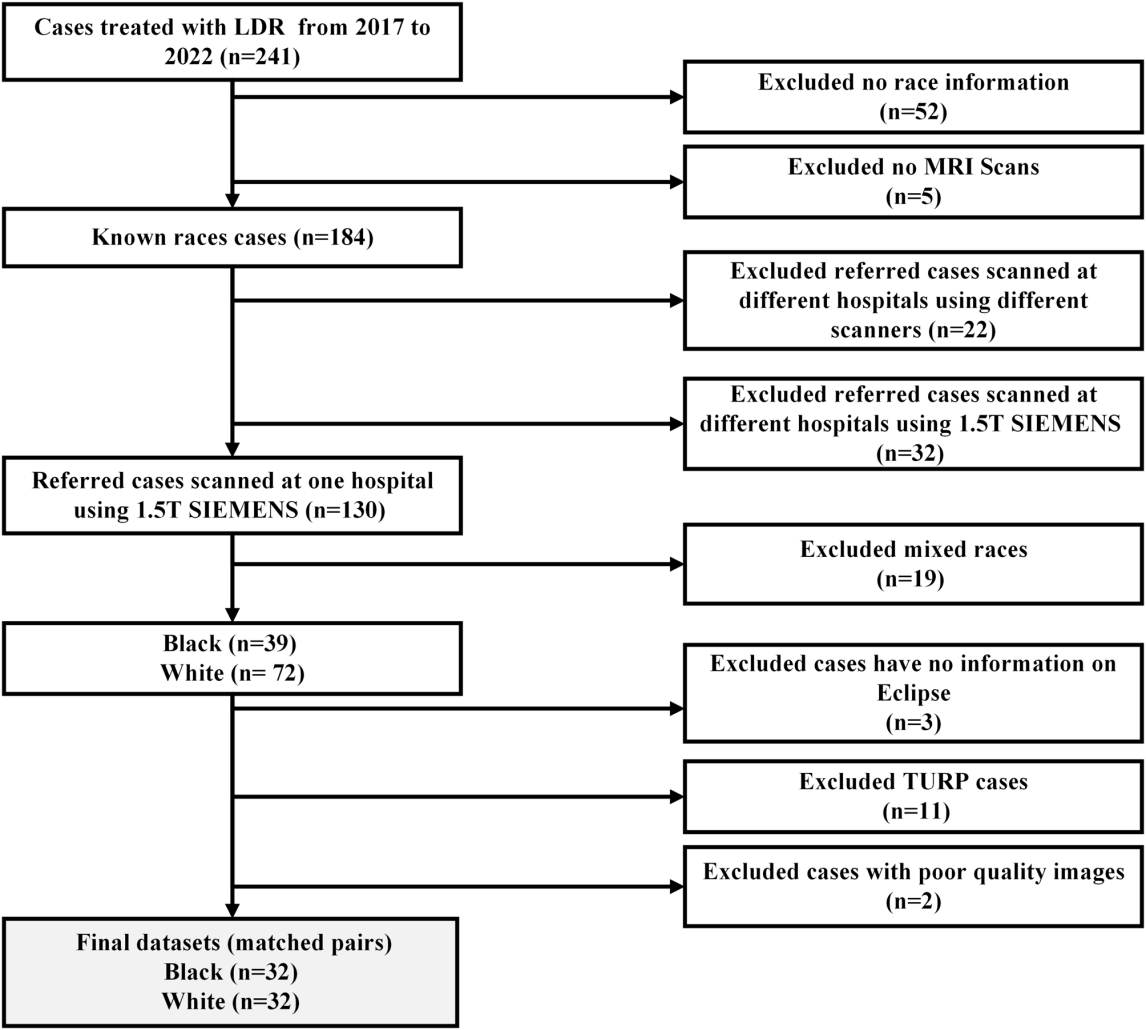
Flowchart illustrating the steps involved in collecting and selecting the in-house (GSTFT) diagnostic MRI dataset. Patients with transurethral resection of the prostate (TURP) or lacking information in the treatment planning system Eclipse were excluded.

The data consisted of pelvis diagnostic MRI scans and race information from patients with low and intermediate risk prostate cancer treated with low dose rate (LDR) brachytherapy (BT) between 2017 and 2022. Figure 1 illustrates the selection process for these data. Note that in selecting the data we ensured that all patients were imaged at the same site and with the same scanner/field strength (Siemens 1.5T) to remove this additional source of distributional shift from the race bias analysis. Only datasets with self-reported race information, specifically White and Black patients, were selected. For White patients, the specific race distribution was: 4% White-Irish, 6% White-English, 66% White-British, 24% White-unspecified. For the Black patients, the distribution was: 38% Black-Caribbean, 2% Black-Ghanaian, 60% Black-unspecified. To control for other possible confounders, we applied a matched pair design approach that randomly selected patients such that each pair of Black/White patients had similar prostate volume (with a tolerance of ± 5 cm^3^) and age (± 10 years). This resulted in 64 patients in total (32 White and 32 Black). All data were pseudonymised using the radiotherapy treatment planning system Eclipse (Varian medical System, Balo Alto-USA) before analysis. Table 1 summarises the patient characteristics of the dataset.

**Table 1:** GSTFT prostate MRI dataset characteristics broken down by race. Median and data range (Min, Max) presented. * refers to a statistically significant difference (p<0.05) based on a Mann-Whitney U test.

Characteristic	White	Black
Age at diagnosis	64 (52,73)*	60.5 (47,74)*
Prostate volume	31 (20,60)	30 (20,65)
BMI	27.2 (21.8, 41.9)	27.7(22.9, 34.9)
Slice thickness	3 (3,3.2)	3 (3, 3.5)
Pixel spacing	0.344 (0.313,0.391)*	0.313 (0.313, 0.375)*
Prostate specific Antigen (PSA)	6.5 (2.17, 28)	7 (2.88, 55.7)
Coil	Body coil	Body coil
MRI sequence	T2W	T2W
TR (ms)	7590 (628–8520)	7590 (678–8500)
TE (ms)	96 (9.9–139)	96 (11–128)
Matrix size	640 (512–640) × 640 (512–640)	640 (640–640) × 640 (640–640)
Scanner	Siemens-1.5T	Siemens-1.5T
# Of Subjects	32	32

For the second experiment (evaluating a model trained with public data), we used five public prostate MRI image datasets for training: PROMISE12^31^, the Initiative for Collaborative Computer Vision Benchmarking (I2CVB)^32^, the Medical Segmentation Decathlon (MSD)^33^, PROSTATEx (version I)^34^, and NCI-ISBI2013^35^. This resulted in a total of 385 subjects (dataset characteristics are shown in Table 2). The same GSTFT clinical data as in the first experiment were used for evaluation.

**Table 2:** The public MRI image databases utilised in the experiments.NA: Not Available. *PROMISE12 was not broken down by scanner field strength in the PROMISE12 challenge website, but most cases were broken down by another work^36–38^

Dataset name	Source	Scanner (field of strength)	Coil	Number of used cases
MSD	Radboud University Medical	NA	NA	32
PROMISE12	Haukeland University Hospital (HK)	Simense (1.5T)	Endorectal	12
PROMISE12	The Beth Israel Deaconess Medical Center	GE (3T)	Endorectal	12
PROMISE12	University College London (UCL)	Siemens (1.5T & 3T)	No	13
PROMISE12	NA*	NA*	NA*	12
PROSTATEx	Radboud University Medical Centre	Siemens (3T)	No	204
NCI-ISBI2013	Boston Medical Center	Philips(1.5T)	Endorectal	30
NCI-ISBI2013	Radboud University Nijmegen Medical Centre	Siemens (3T)	Surface	30
I2CVB	Chu Dijon Bocage	Siemens (3T)	Body	19
I2CVB	Ressonancia Girona	GE (1.5T)	Body and Atdtorso coil	21

### Data preparation

For the GSTFT clinical dataset, all images used had ground truth prostate segmentations initially produced by a senior clinical fellow(1 year contouring experience) and then reviewed by an expert clinical oncologist (25 years contouring experience) with a random selection representing 10% of the total cases also reviewed by a radiologist (15 years contouring experience). The segmentations were performed manually using the treatment planning system Eclipse. All segmentations underwent some degree of editing after review. Note that this manual segmentation process was necessary as there were no clinically-created segmentations for MRI scans. This is because co-registration and subsequent segmentation of the prostate from MRI is not routinely performed in the radiotherapy planning workflow at GSTFT, although there are currently plans for a transition towards their use for this particular application. As all patient data were pseudonymised prior to segmentation and exported from Eclipse, the annotator was blinded to the race information. The segmentations exported from Eclipse were formatted as continuously spaced contour points that define the boundary of a region of interest. To make these contours suitable for use in training the DL segmentation model, we used the rt-util^39^ Python library to convert them into binary masks. All images and labels were converted from DICOM to NIFTI format before use in the experiments as nnU-net requires the input images/labels to be in NIFTI format.

As stated earlier, this study incorporated five publicly available prostate MRI datasets, each with differing levels of transparency regarding their ground truth segmentations. All the datasets came with manual ground truth segmentations of the prostate, except MSD, where only the peripheral zone (PZ) and transitional zone (TZ) labels were provided. Note that, in radiation oncology, the whole prostate gland is irradiated^40^. Thus, PZ and TZ were merged to produce whole prostate gland labels for MSD. Information about the expertise of the annotators was not publicly disclosed, except for PROMISE12 and NCI-ISBI2013. For PROMISE12, the annotations were provided by experienced observers (radiologists, residents, or image analysis researchers) and verified by radiologists with more than 6 years experience. For NCI-ISBI2013, they were produced by two clinical doctors with undefined experience. Like the GSTFT data, all images and labels in the public datasets were converted into NIFTI format before use in the experiments.

### Experiments

We employed the nnU-Net DL segmentation model^14^ with the 2D configuration as suggested by the nnU-Net developers since prostate MRI images are typically anisotropic. nnUNetTrainerV2 was used with the default settings for all experiments except for the training epochs, which were set to 500 epochs to make the training faster. Two experiments were conducted, which are explained in the subsections below. All experiments were carried out on two local machines using A6000 48 GB GPUs and the Ubuntu 18.04.6 LTS operating system. The PyCharm virtual environment was used with PyTorch version 1 1.9.0 + cu111 for Machine 1 and 1.8.1 + cu111 for Machine 2.

- Experiment 1: Controlled race bias investigation.

In this experiment, we created five training datasets from the GSTFT data in which the proportions of the subjects’ races varied systematically: 0%/100% White/Black, 25%/75% White/Black, 50%/50% White/Black, 75%/25% White/Black and 100%/0% White/Black. For each model, we performed a four fold cross-validation maintaining the matched pairs for each left-out fold, resulting in 24 subjects for training and 16 subjects (8 matched pairs) for testing per fold. Since nnU-Net does not use the validation set for hyperparameter selection or model selection, this cross-validation process enabled evaluation on all 64 subjects as independent test data. Our selection of subjects for the training datasets was random, ensuring that only the exact proportion was taken from each group, regardless of whether the matched pairs were present or not. However, the evaluation set did contain only matched pairs. To ensure the same training set size for all models (i.e., with different race ratios), from the 48 patients in the training folds (which were 24 White and 24 Black), we randomly selected 24 patients of the required race for model training for the 0%/100% White/Black and 100%/0% White/Black models. For the other race ratios, patients were randomly selected to ensure the required ratio, resulting in a total training set size of 24 subjects for each model.

**Fig. 2:**
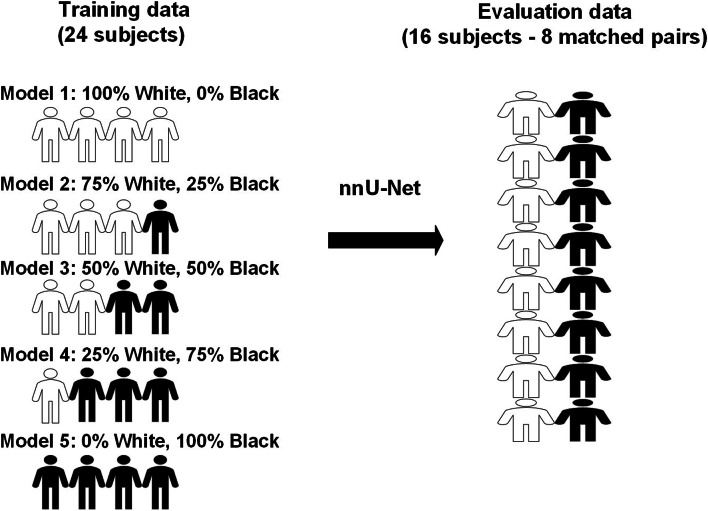
Training and evaluation dataset split for assessing race bias in DL-based prostate MRI segmenta tion.

An illustration of the training and evaluation splits is shown in Fig. 2.

- Experiment 2: Training on unknown race public data.

In this experiment, a five fold cross-validation was performed on the public training dataset, resulting in 5 trained models (for each fold, nnU-Net splits the data into 20% for validation and 80% for training). We applied an ensemble of the 5 models on the full GSTFT dataset for evaluation. Note that the use of cross validation in this experiment aimed to improve model robustness whereas in Experiment 1 it was to maximise data use.

### Evaluation and statistical analysis

In both experiments, model performance was evaluated using the Dice similarity coefficient (DSC), surface Dice Similarity Coefficient (sDSC), Hausdorff distance (HD) and 95% HD^41,42^. p-values for Mann–Whitney U tests were computed to test for statistically significant differences (significance level 0.05) between White and Black patients in the test set. As an exploratory study we did not correct for multiple testing. To assess the impact of potential confounders, we performed multivariate linear regression on the quantitative results for the 0% Black/100% White model which showed possibly biased performance in the first experiment. Selected variables analysed, in addition to race, were age, presenting prostate-specific antigen (PSA), prostate volume, MRI slice thickness, body mass index (BMI), MRI voxel spacing. Additionally, Spearman’s correlation coefficient was used to measure the relationship between the percentage of Black training subjects and (i) the median DSC for both Black and White test subjects, (ii) the interquartile range of DSC for Black and White subjects and (iii) the median difference in DSC between Black and White matched pairs. We also applied linear mixed-effects modelling to investigate whether the difference in the DSC between Black and White subjects was dependent on the percentage of Black subjects in the training data. The MATLAB^43^ software was used to perform the statistical analysis.

## Results

**Fig. 3:**
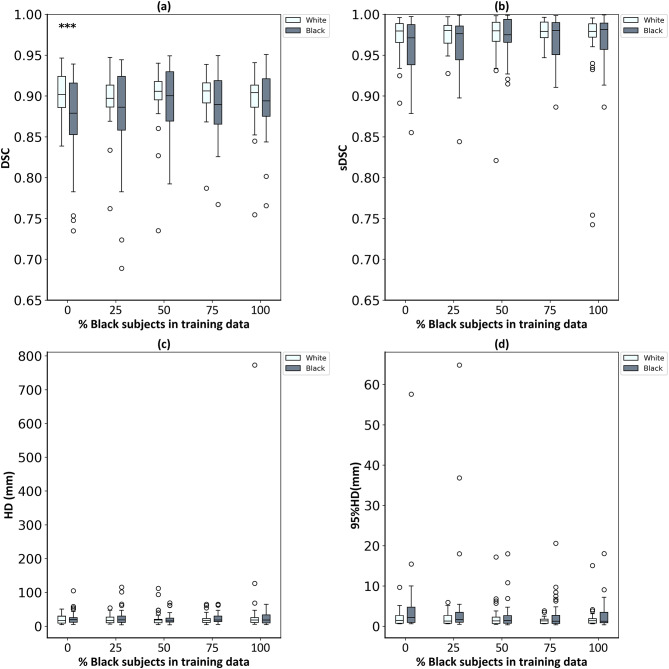
The performance of DL segmentation models on 32 matched pairs of White/Black race. (a) DSC, (b) sDSC, (c) HD mm, (d) 95% HD mm. p-values computed using Mann–Whitney U tests at 0.05 significance level (*** = statistically significant at 0.05 significance level).

The results of the first experiment are shown in Fig. 3. The best results for both races were obtained when the model was trained using an equal proportion of White and Black subjects, based on DSC, HD, and 95% HD. The Mann–Whitney U test p-values for comparing DSCs between Black and White subjects were all greater than 0.05, with the exception of the 0% Black/100% White trained model (p = 0.03), which showed higher DSCs for the White subjects. The Spearman’s correlation coefficients presented in Table 3 showed a significant negative correlation between the number of Black subjects in the training set and the interquartile range of DSC scores for Black subjects (rho = -1, p < 0.05).

**Table 3:** Spearman’s correlation coefficients between the percentage of Black training subjects and (i) IQR- B: the interquartile range of Black subjects’ DSCs, (ii) IQR-W: interquartile range of White subjects’ DSCs, (iii) Med-B: median DSC of Black subjects, Med-W: median DSC of White subjects, (v) Med B-W diff: median of differences in DSC between Black and White pairs. * = Correlation is significant at the 0.05 level.

	IQR-B	IQR-W	Med-B	Med-W	Med B-W diff
Rho	-1.00*	-0.70	0.70	0.60	0.20
p-value	0.02*	0.23	0.23	0.35	0.78

The results of the multivariate linear regression between DSC values and covariates for the 100%/0% White/Black model (which were highlighted as possibly biased in Fig. 3a) are shown in Table 4. The only variables that could explain the bias found (p < 0.05) were race, BMI and slice thickness.

**Table 4:** Multivariate linear regression associations between DSC and possible covariates for the 100%/0% White/Black model. * = Association is significant at the 0.05 significance level.

Confounder	Effect (*β*)	DSC (p-value)
Race	0.04	0.00*
Age	-0.00	0.17
PSA	0.00	0.20
Volume	-0.00	0.24
Slice thickness	-0.17	0.05*
Pixel Spacing	0.20	0.50
BMI	0.00	0.01*

The DSCs of the five models for Black and White subjects were further analysed using a linear mixed-effects model with race, percentage of Black subjects in the training data, and their interaction as fixed effects (random intercepts by cohort). The DSC for Black subjects increased with percentage of Black subjects in the training data (*β* = 0.00022 per 1% increase, p = 0.008), corresponding to an improvement of approximately 0.0055 DSC for every 25% increase in Black subjects in training. Importantly, the interaction was statistically significant (*β* = 0.00024, p = 0.041), indicating that the higher DSC observed in White subjects decreased as the percentage of Black subjects in the training data increased. Specifically, the White–Black performance gap narrowed by about 0.006 DSC for every 25% increase in Black subjects in training.

**Fig. 4:**
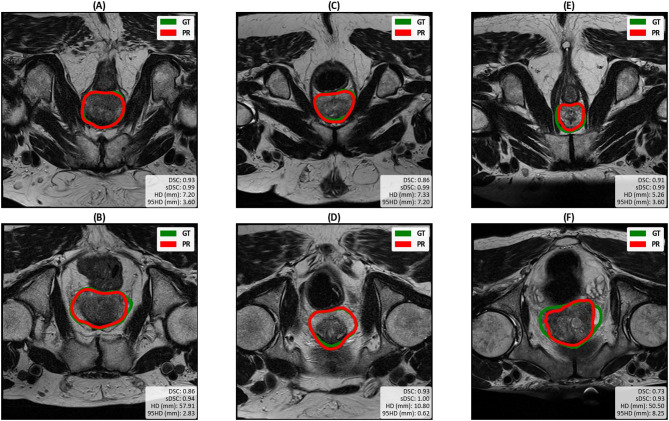
The performance of nnU-Net trained on 100%/0% White/Black subjects from the GSTFT dataset. The top row shows the White test subjects, and the bottom row shows the (paired) Black test subjects. GT: Ground truth segmentation, PR: predicted segmentation.

To enable a qualitative assessment of the race bias, we visualised the DL-generated segmentations of the 100%/0% White/Black model. Figure 4 shows the predicted and ground truth segmentations for three White/Black matched pairs. The axial slice displayed was chosen by selecting the slice where the prostate is located for all the cases. The images do not appear to show any specific pattern in errors for either race, but a noticeable drop in accuracy can be observed on one of the Black subjects (Fig. 4F) compared to its White matched pair (Fig. 4E).

**Fig. 5:**
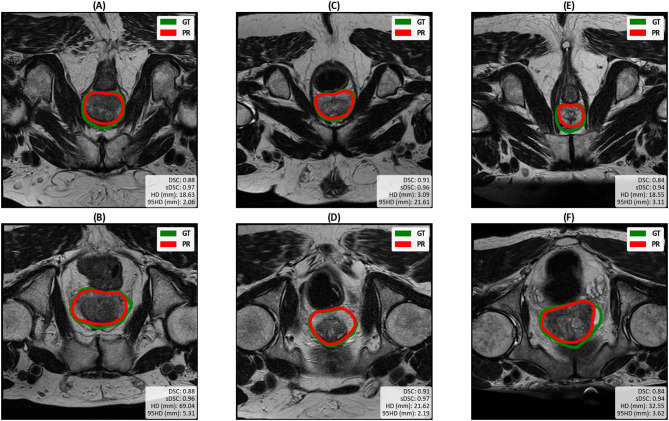
The performance of nnU-Net trained on the public datasets. The top row shows the White test subjects, and the bottom row shows the (paired) Black test subjects. GT: Ground truth segmentation, PR: predicted segmentation.

The performance of the model trained using public data when tested on White and Black patients from the full GSTFT dataset is shown in Table 5. The median and interquartile ranges of DSC, sDSC, HD and 95% HD for the Black and White patients’ predicted segmentations were 0.87 (0.07)/ 0.85 (0.06), 0.96 (0.04)/0.94 (0.07), 23.66 (14.55)/27.23 (19.94) and 3.15 (3.48)/3.56 (3.34), respectively. There was no statistically significant difference in performance between the races, except for sDSC. Qualitative results of the model trained using public data and tested on the same GSTFT cohort are shown in Fig. 5.

**Table 5:** The median and interquartile ranges (IQR) of predicted segmentation DSCs, sDSCs, HDs and 95% HDs of the model trained using unknown race public prostate MRI datasets, for Black and White patients from the GSTFT dataset. * = Statistically significant difference based on Mann–Whitney U test at 0.05 significance level.

	DSC		sDSC		HD (mm)		95% HD (mm)	
	Black	White	Black	White	Black	White	Black	White
Median	0.87	0.85	0.96	0.94	23.66	27.23	3.15	3.56
IQR	0.07	0.06	0.04	0.07	14.55	19.64	3.48	3.34
p-value	0.17		0.02*		0.37		0.20

## Discussion

Recently, there has been movement towards greater regulation of artificial intelligence (AI) models and model bias is a key component of the proposed legislation/guidelines. For example, the recent guidelines issued by the National Institute for Health and Care Excellence (NICE) in the UK require that AI technology developers should consider equality when implementing AI clinically and training data demographic information should be provided^29^. The recent update to the TRIPOD-AI guidelines has fairness embedded in a number of its reporting recommendations^30^.

Our work has investigated race bias in DL segmentation models for prostate MRI in two ways. First, we systematically varied the White/Black race imbalance of the training dataset in a controlled experimental setting. Second, we trained a model on public data (of unknown race). In both experiments, evaluation was performed on a clinical dataset of paired White/Black patients. The controlled experiment showed that the DL segmentation model showed signs of possible race bias when the model was trained with highly imbalanced data (100%/0% White/Black). However, the effect was relatively small (a DSC difference of 0.02) and would not have been statistically significant with correction for multiple testing (p-value = 0.03, adjusted significance level using Bonferroni correction = 0.05/5 = 0.01). Nevertheless, we believe this finding to be worthy of further investigation and the lack of statistical significance may be Type II error, which should be investigated in future work with a larger test set size. The multivariate linear regression analysis indicated that patient race, BMI and image slice thickness were the only variables that could explain this observed difference. However, the slice thickness was the same for all 64 subjects except for two subjects, which might have led to this low p-value. For BMI, while it was statistically significant (p = 0.01), its effect size (*β* = 0.00) was considerably smaller than that of race (*β* = 0.04, p = 0.00), indicating that race had a more robust association with DSC. There was a noticeable reduction in the variability (i.e. interquartile range) of Black subjects’ DSCs as the number of Black subjects in the training set increased. The best performance for both races was achieved when training with equal proportions of White and Black patients, based on DSC, HD, and 95% HD.

Perhaps surprisingly, the first experiment showed approximately constant performance on White subjects regardless of the imbalance in the training data, whereas performance for Black subjects did vary by the level of imbalance. A linear mixed-effects model analysis of these trends showed a statistically significant improvement in Black subjects’ DSC of 0.0055 for every 25% increase in Black subjects in the training data (p = 0.008). Similarly, the White–Black performance gap narrowed by 0.006 DSC for every 25% increase in Black subjects in training (p = 0.041). We speculate that this may be caused by differences in the variation in image characteristics of the MRI data between and within the two race groups. For example, if the Black group had more within-group diversity this would cause the model trained using only Black subjects’ data to have more robustness to distributional shift (such as evaluating it on White subjects). Conversely, if the White group had less within-group diversity it would not perform so well in the presence of distributional shift (e.g. on Black subjects). Consequently, the White-trained model would benefit more from the introduction of patients from the other race group into the training set. This agrees well with the findings of Čevora et al.^44^, who reported that sometimes a model trained using entirely data from one demographic group achieved better performance when evaluated on a different group than when evaluated on the same group. For example, they found that model performance on segmenting female left kidneys was better when the training set was only from male subjects compared to training with an entirely female training set. This highlights the complexity of performance bias and the need for better understanding of demographic distributional differences in medical image datasets.

In our work, the specific cause of the distributional shift between White and Black patients’ MRI data remains open to speculation. However, one study has reported that anatomical variations of pelvis parameters might be associated with race^45^, Zárate-Kalfópulos et al. compared the pelvic parameters (lumbar lordosis, pelvic incidence, pelvic tilt, and sacral slope) of healthy Mexican volunteers with Caucasians and Asians and found statistically significant differences in these parameters between groups. Although in our case the target of the segmentation model is the prostate, DL models’ internal representations are influenced by contextual information and so model bias could still result from pelvic anatomical differences. Future work should also focus on investigating the source(s) of the distributional shift between races in prostate MRI.

A possible limitation of this work is that the ground truth segmentations for the GSTFT data were produced by a single annotator, which could in theory have impacted the results. However, we emphasise that the annotator was blinded to the race of the subjects, and furthermore that all segmentations were reviewed by a clinical oncologist with a subset reviewed by a radiologist. Therefore, we believe that the ground truth segmentations were of high quality and the effects of individual annotation style did not impact our results greatly.

The performance of the model trained on public heterogeneous data showed no statistically significant difference in performance between White and Black patients, except for sDSC. This might indicate that the training data used was diverse enough to reduce the bias. However, one limitation of this finding is that the public datasets’ race information was not provided, so the racial heterogeneity of the training data was not known. In our dataset, we focused on White and Black subjects as they represented 85.38% of the races within the dataset available to us that met the our study criteria. However, we acknowledge that our dataset was relatively small, and therefore further validation on a larger cohort of different races is needed.

Another limitation is that the GSTFT dataset contained some post-biopsy MRIs, whereas the public dataset was all pre-biopsy. Specifically, for 16 out of the 64 patients (9 Black and 7 White), post-biopsy scans were used (with a median gap of 4 months from biopsy to MRI) as a result of some patients being on an active surveillance program. It can be argued that the potential small differences in appearance of the prostate between pre- and post- biopsy MRIs could impact model performance and therefore may represent a limitation to our work. However, the post-biopsy scans represented only 25% of the total cases, with nearly equal proportions across the White and Black cohorts. Therefore, we believe that any impact would be small. Using these scans was a practical decision we took based on data availability and further work is required to investigate if the race bias effect persists in experiments using only post-biopsy MRIs.

To comply with the requirements of the nnU-Net pipeline, all images were converted from the original DICOM format into NIFTI format. This conversion had no adverse effect and preserved the image’s affine information. However, if conversion is mishandled, it can impact calculation of boundary distance-based metrics such as HD and also impact subsequent clinical tasks such as treatment planning and dosimetry.

To the best of our knowledge, no previous work has investigated race bias in DL based prostate segmentation. The most closely related work in radiation oncology is by McQuinlan et al.^46^, who investigated possible geographic bias in head and neck computed tomography (CT) segmentation. This work did report some differences in quantitative segmentation metrics, but these were mostly explained by differences in structure volumes impacting the metrics. This work did not focus explicitly on race. A previous work^47^ has shown that the ratio between transition zone volume and total prostate volume (called the transition zone index) was significantly higher in African American than Hispanic and Caucasian men. Future work should consider using transitional/peripheral zone size as a possible confounder that could affect the model performance differences between races.

To conclude, in this work, we have shown for the first time a possible race bias effect in DL-based prostate MRI segmentation. Specifically, we have shown that the performance gap between Black and White subjects is dependent on the proportion of Black subjects in the model training data. This finding is important as race bias may also affect other applications of DL in prostate MRI analysis, such as tumour detection and classification^48,49^. Based on this, we emphasise the importance of (i) transparency in reporting of demographic information for DL model training data, (ii) open reporting of DL model test performance for different demographic groups and (iii) training DL segmentation models with demographically diverse training data with appropriate ethical and legal measures in place. However, healthcare equity should not come at the expense of the patient privacy. Whilst reporting of summaries of race (and other demographic) information is important, this information at the subject level should be kept secure, and only authorised parties should have access to it. Furthermore, it is important that if this information is used, it should not be used for discriminatory practices. Clear protocols compliant with laws and regulations^50–52^ should be in place to safeguard patient data and ensure privacy and confidentiality are maintained throughout the research process.

## Data Availability

Part of the datasets used in our study are publicly available, which are PROMISE12 (PROMISE12: https://promise12.grand-challenge.org/Details/), the Initiative for Collaborative Computer Vision Benchmarking (I2CVB) (I2CVB: https://i2cvb.github.io/), the MSD (MSD: http://medicaldecathlon.com/), the PROSTATEx (PROSTATEx: https://prostatex.grand-challenge.org/), and NCI-ISBI2013 (NCI-ISBI2013: https://www.cancerimagingarchive.net/analysis-result/isbi-mr-prostate-2013/). The rest of the dataset are in-house data that is available from Guy’s and St. Thomas’ NHS Foundation Trust. Restrictions apply to the availability of these data, which were used under license [Guy’s Cancer Cohort REC Reference: 18/NW/0297] for the current study, and so are not publicly available.
